# REDEFINING OBESITY: A RATIO OF FAT AND MUSCLE MASS COMPARED TO BODY MASS INDEX IN OLDER ADULTS

**DOI:** 10.1093/geroni/igad104.3560

**Published:** 2023-12-21

**Authors:** Kworweinski Lafontant, Ladda Thiamwong, Jeffery Stout, Joon-Hyuk Park, Rui Xie, David Fukuda

**Affiliations:** University of Central Florida, Orlando, Florida, United States; University of Central Florida, Orlando, Florida, United States; University of Central Florida, Orlando, Florida, United States; University of Central Florida, Orlando, Florida, United States; University of Central Florida, Orlando, Florida, United States; University of Central Florida, Orlando, Florida, United States

## Abstract

Body composition is an important determinant of physical fitness and overall health in older adults. Body mass index (BMI) is currently the standard used to categorize clinical thresholds for body composition (underweight, normal weight, overweight, obese). However, BMI does not account for key aspects of body composition like skeletal muscle mass (SMM) and fat mass (FM). This can lead to misclassification and inaccurate inferences about metabolic health. A FM/SMM ratio may better capture age-related changes in body composition like sarcopenia and obesity, but it is unknown whether FM/SMM correlates more closely with muscularity and adiposity than BMI. This cross-sectional study assessed 103 older adults (16 men, 87 women; mean age 75.7 ± 7.19 years; BMI 26.8 ± 5.22 kg/m2) for SMM, FM, and body fat percentage (BF%) using bioelectrical impedance analysis (InBody S10). BF% was more highly correlated with FM/SMM (r = 0.979, p < 0.001) than BMI (r = 0.702, p < 0.001), and a Fisher’s R-to-Z analysis indicates that the difference is significant (95% CI [0.826, 0.926], z = 12.217, p < 0.001). However, SMM showed weak correlation with both FM/SMM (r = -0.306, p = 0.002) and BMI (r = 0.349, p < 0.001). FM/SMM may better detect changes in adiposity compared to BMI, suggesting utility as an obesity measure. Further research should examine the longitudinal validity of FM/SMM in older adults and its ability to predict metabolic and musculoskeletal health.

